# Ultraviolet Photodissociation for Non-Target Screening-Based Identification of Organic Micro-Pollutants in Water Samples

**DOI:** 10.3390/molecules25184189

**Published:** 2020-09-12

**Authors:** Christian Panse, Seema Sharma, Romain Huguet, Dennis Vughs, Jonas Grossmann, Andrea Mizzi Brunner

**Affiliations:** 1 Functional Genomics Center Zurich, University of Zurich/ETH Zurich, Winterthurerstrasse 190, CH-8057 Zürich, Switzerland; cp@fgcz.ethz.ch (C.P.); jg@fgcz.ethz.ch (J.G.); 2 SIB Swiss Institute of Bioinformatics, Quartier Sorge-Batiment Amphipole, 1015 Lausanne, Switzerland; 3 Thermo Fisher Scientific, San Jose, CA 95134, USA; seema.sharma@thermofisher.com (S.S.); romain.huguet@thermofisher.com (R.H.); 4 KWR Water Research Institute, P.O. Box 1072, 3430 BB Nieuwegein, The Netherlands; dennis.vughs@kwrwater.nl

**Keywords:** mass spectrometry, non-target screening, ultraviolet photodissociation, higher-energy collisional dissociation, organic micropollutants, water quality, small molecule fragmentation, cheminformatics, data analysis

## Abstract

Non-target screening (NTS) based on the combination of liquid chromatography coupled to high-resolution mass spectrometry has become the key method to identify organic micro-pollutants (OMPs) in water samples. However, a large number of compounds remains unidentified with current NTS approaches due to poor quality fragmentation spectra generated by suboptimal fragmentation methods. Here, the potential of the alternative fragmentation technique ultraviolet photodissociation (UVPD) to improve identification of OMPs in water samples was investigated. A diverse set of water-relevant OMPs was selected based on k-means clustering and unsupervised artificial neural networks. The selected OMPs were analyzed using an Orbitrap Fusion Lumos equipped with UVPD. Therewith, information-rich MS2 fragmentation spectra of compounds that fragment poorly with higher-energy collisional dissociation (HCD) could be attained. Development of an R-based data analysis workflow and user interface facilitated the characterization and comparison of HCD and UVPD fragmentation patterns. UVPD and HCD generated both unique and common fragments, demonstrating that some fragmentation pathways are specific to the respective fragmentation method, while others seem more generic. Application of UVPD fragmentation to the analysis of surface water enabled OMP identification using existing HCD spectral libraries. However, high-throughput applications still require optimization of informatics workflows and spectral libraries tailored to UVPD.

## 1. Introduction

### 1.1. Challenges of Monitoring Drinking Water Quality

Reliable identification of organic micro-pollutants (OMPs) in drinking water and its sources is essential to risk assessment and prediction of the behavior of a substance in the environment and during water treatment. Non-target screening (NTS) based on the combination of liquid chromatography coupled to high-resolution mass spectrometry (LC-HRMS/MS) has become the key method to identify OMPs in water samples, as it has the potential to detect all ionizable compounds that are amenable to the selected chromatographic separation, within a defined mass range [1].

The unambiguous identification of an OMP from NTS data relies on the accurate mass and isotopic pattern from the full scan MS1 spectrum to determine the elemental formula of the compound. The addition of MS2 fragmentation spectral data then allows to determine the molecular structure, given that the fragmentation event causes reproducible bond cleavages that result in diagnostic and interpretable fragment ions representative of the structure of the molecule. The MS2-based structural identification typically relies on matching of the experimental spectrum with entries in spectral libraries or *in silico* predicted fragmentation spectra. For compounds that show poor fragmentation spectra generated by higher-energy collisional dissociation (HCD) fragmentation, the fragmentation technique routinely applied in Orbitrap based NTS, confident structural elucidation often remains elusive. Alternative fragmentation techniques that cause different bond cleavages may remedy structural elucidation in these cases.

### 1.2. Interpreting Fragmentation Spectra from Ultraviolet Photodissociation

Ultraviolet photodissociation (UVPD) is a fragmentation technique achieved with a UV laser. Its main application to date is protein characterization with proteomics and intact protein MS. However, it also allows structural elucidation of small molecules that cannot be identified by HCD alone [2]. For instance, UVPD was shown to facilitate characterization of various lipid classes [3], to generate unique fragments or enhance detection of kinetically unfavorable fragments of flavonoids, phenylpropanoids and chalconoids [4,5].

In the Orbitrap Fusion Lumos mass spectrometer, a Q3 series passively Q-switched Nd:YAG laser (CryLaS GmbH) that outputs the 5th harmonic at 213 nm is interfaced to the rear of the ion trap, in the low pressure cell of which the UV photoactivation occurs [6]. The laser pulse energy is a 1.5 ± 0.2 μJ/pulse at 2.5 kHz repetition rate. With 450 ± 200 μm, including the divergence at the center of the ion trap, the beam diameter is slightly larger than the simulated ion cloud diameter at normal AGC targets and no focusing optics are required. With the incorporation of this compact and robust solid state laser into a commercial instrument, UVPD could be routinely implemented in NTS workflows.

To date, however, UVPD fragmentation has not been applied to the NTS-based monitoring of small molecules. This is due to the fact that apart from the compounds mentioned above, little is known about UVPD fragmentation pathways of small molecules, the kinetics of fragment formation, and the influence of reaction time on fragmentation patterns. Moreover, as it is a novel fragmentation technique in the small molecule field, spectral library entries with UVPD spectra are still lacking, and the usability of HCD spectra for spectral matching of UVPD spectra remains to be explored. Furthermore, the aptness of *in silico* prediction algorithms for the prediction of UVPD spectra has not yet been demonstrated. Combinatorial prediction algorithms that do not apply any rules of fragmentation, but use a bond dissociation approach, could potentially be used for UVPD data.

### 1.3. UVPD Fragmentation for Water Quality Monitoring

Here, the potential of the fragmentation technique UVPD to improve the structural identification of small molecules, in particular OMPs in water samples, was evaluated using the Orbitrap Fusion Lumos Tribrid [6]. After application of a cheminformatics strategy to select water relevant OMPs that cover a wide chemical space and development of a data analysis workflow in R, HCD and UVPD fragmentation patterns of selected OMPs could be characterized and compared. The two fragmentation techniques generated both unique and common fragments, demonstrating that some fragmentation pathways are specific to the respective fragmentation method whilst others seem more generic. Application to environmental water samples showed that HCD spectral libraries can be used for UVPD based OMP identification, in particular when high collision energy (CE) spectra are available. However, to increase successful feature annotation of NTS data with UVPD fragmentation, UVPD spectra need to be added to spectral libraries.

## 2. Results and Discussion

### 2.1. Proof of Principle: Manual Spectral Interpretation of Single Compounds

To investigate the potential of UVPD for OMP identification, the three compounds triadimenol, gemfibrozil and sucralose were selected as model compounds. These compounds are both relevant for the water sector and known to not fragment well using standard HCD fragmentation settings, i.e., CEs ranging from 20 to 50 (arbitrary units). As little was known about UVPD fragmentation pathways of these OMPs, we applied the combinatorial prediction algorithm of MetFrag [7], which does not rely on fragmentation rules, but uses a bond dissociation approach to predict potential fragments and matches these to the experimentally observed.

**Figure 1:**
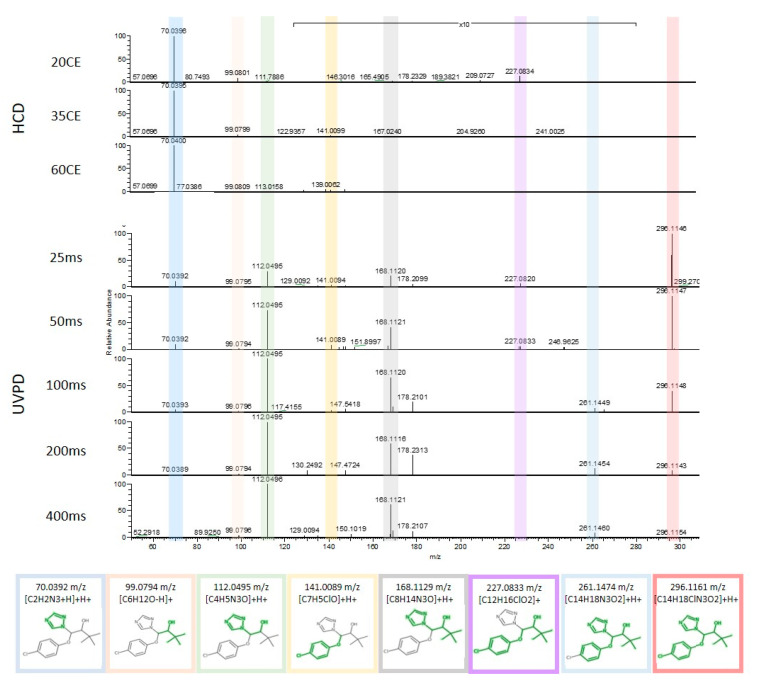
Comparison of higher-energy collisional dissociation (HCD) (top) and ultraviolet photodissociation (UVPD) (bottom) fragmentation spectra of the fungicide triadimenol acquired with 20, 35 and 60 collision energy (CE) and 25, 50, 100, 200 and 400 ms reaction time. Annotated fragments are highlighted in color. The *m/z* range 130–280 is 10× enlarged.

UVPD provided unique fragmentation information for structural elucidation of triadimenol, a fungicide that can be found in drinking water sources (Figure 1). HCD fragmentation at 35CE, the CE range typically used in NTS experiments, resulted in a predominant fragment at 70 *m*/*z*, a minor fragment at 99 *m*/*z* and a low intensity fragment (see 10× zoom-in) at 141 *m*/*z*. These fragments could be assigned to the *in silico* predicted fragments [C_2_H_2_N_3_ + H] + H^+^, [C_6_H_12_O-H]^+^ and [C_7_H_5_ClO] + H^+^. In contrast, UVPD fragmentation led to more and different fragment ions. The peaks detected with HCD were also detected in the UVPD fragmentation spectra when using shorter reaction times (25 to 100 ms), but decreased with increasing UVPD reaction times. Concurrently, peaks at 112, 168, and 261 *m*/*z* increased with increasing reaction times. These could be matched to the *in silico* predicted fragments [C_4_H_5_N_3_O] + H^+^, [C_8_H_14_N_3_O] + H^+^, and [C_14_H_18_N_3_O_2_] + H^+^. A fragment at 227 *m*/*z* was detected with UVPD at short reaction times, i.e., 25 to 50ms only and could be matched to [C_12_H_16_ClO_2_]^+^. These promising results showed that UVPD could lead to informative spectral information of an OMP that did not fragment well with HCD, and that the *in silico* prediction using MetFrag could successfully be applied for UVPD spectral annotation.

Next, UVPD fragmentation of OMPs that ionize in negative ionization mode were investigated, starting with the pharmaceutical gemfibrozil. Two peaks could be annotated in the HCD spectrum with 35CE and UVPD spectrum with 25ms reaction time, namely [C_8_H_9_O]^−^ at 121 *m*/*z*, and [C_7_H_13_O_2_-H]-H^-^ at 127 *m*/*z* (see Figure S1a). In the UVPD spectra with longer reaction times, only the 121 *m*/*z* peak could be matched. The predominant UVPD peak at 112 *m*/*z* increased with reaction times. This peak was also present in the HCD spectrum. However, it could not be matched to an *in silico* predicted fragment mass, nor could any of the other UVPD peaks. The base peak in all UVPD spectra was the precursor ion, the absolute intensity of which decreased with increasing reaction times. As observed previously with UVPD of lower charged negative DNA ions, this could be due to an electron detachment-induced charge reduction [2]. Electron detachment dissociation usually involves two or more negatively charged species. For single ion negative UVPD, the mechanism for electron detachment may be more favorable than fragment generation. However, these data were based on 193 nm UVPD, and whether 213 nm UVPD would have the same effects remains unknown.

As a second OMP analyzed in negative ionization mode, the artificial sweetener sucralose was fragmented. With the standard HCD fragmentation with 35CE, six peaks could be annotated (see SI Figure 1b). Four of the annotations, i.e., [C_2_H_4_O_2_]-H^−^ at 59 *m*/*z*, [C_3_H_5_O_2_-H]-H^−^ at 71 *m*/*z*, [C_3_H_5_O_2_]^−^ at 73 *m*/*z* and [C_3_H_5_O_3_-H]-H^−^ at 87 *m*/*z*, were low mass range fragments only detected using HCD fragmentation. The other two, [C_4_H_7_O_3_-H]-H^−^ at 101 *m*/*z* and [C_6_H_10_O_4_-2H]-H^−^ at 143 *m*/*z*, were present in both HCD and UVPD spectra with 50, 100, 200 ms and—in the latter—400 ms reaction time. At 25 ms, the species, [C_6_H_10_O_4_-H]-H^−^ at 144 *m*/*z*, was present instead. At 400 ms reaction time, the 143 *m*/*z* peak was the only one that could be annotated in an overall noisy low intensity fragmentation spectrum. Corresponding to what was observed for triadimenol, long UVPD reaction times negatively affected spectral quality as well as the duty cycle in the case of sucralose.

In the spectra of all shorter reaction times, i.e., 25 to 200 ms, the fragments [C_6_H_10_O_5_-2H]-H^−^ at 159 *m*/*z* and [C_6_H_9_Cl_2_O_3_]-H^−^ at 197 *m*/*z* could be annotated; at lower reaction times also [C_6_H_9_Cl_2_O_3_]^−^ at 198 *m/z* and [C_6_H_9_Cl_2_O_3_-H]-H^−^ at 196 *m*/*z*. At 50 ms, another annotated fragment, [C_6_H_10_ClO_5_-H]-H^−^ at 195 *m*/*z*, was present. This reaction time resulted, thus, in the most informative spectra, in particular in combination with the HCD spectrum of low mass range annotated fragments.

The UVPD fragmentation data of the three model compounds of which one ionized in positive and two in negative ionization mode suggested that UVPD could facilitate structural elucidation of some OMPs for which HCD spectra did not contain enough information. While fragmentation of the positively charged triadimenol led to more fragments and higher fragment intensities with UVPD compared to HCD, the negatively charged compound gemfibrozil fragmented poorly with both HCD and UVPD, and UVPD fragmentation of sucralose resulted in complementary fragments to HCD.

### 2.2. Selection of Reference Standards Based on Clustering

**Figure 2:**
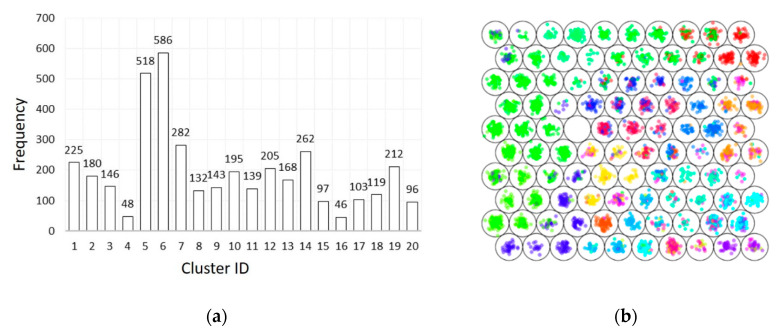
Selection of organic micro-pollutants (OMPs) by two complementary approaches: (**a**) k-means clustering of Pubchem extended fingerprints; (**b**) self-organizing map. Compounds are colored according to the k-means cluster number.

To further investigate the applicability of UVPD for OMP identification, a representative selection of compounds regarding their distribution in the chemical space and water relevance was made. First, a k-means clustering was performed using Pubchem extended fingerprints, resulting in 20 defined clusters (Figure 2a). The molecular discrimination of these clusters was confirmed using the unsupervised artificial neural network self-organizing maps (SOM, Figure 2b). In the SOM, the selected compounds were colored according to their k-means cluster number. As compounds of the same color are in close vicinity in the SOM, this shows that the different clusters successfully separated these compounds.

Depending on in-house reference standard availability, one to four compounds were selected per cluster for fragmentation experiments, apart from cluster 19, for which no standard was available. In addition, a selection of disinfection by-products known to be relevant for drinking water treatment was added to the set of compounds, to be fragmented by HCD and UVPD.

### 2.3. An R-Based Data Analysis Workflow and Shiny Application Interface to Explore (Novel) Fragmentation Techniques

To enable high throughput data analysis of the LC-HRMS data including UVPD fragmentation spectra, an R-based workflow was developed that takes the extracted ion chromatogram (XIC) of a given compounds based on its simplified molecular-input line-entry specification (SMILES), determines the peak apex and extracts the corresponding retention time (RT). The three MS2 spectra with highest intensity neighboring the apex were used to match experimental spectra with *in silico* predicted fragmentation spectra of the given SMILES. 

For a user friendly output and to support exploratory data analysis [8], a Shiny application based interface was created to further examine the data (https://CRAN.R-project.org/package=shiny). The Shiny application is provided with the ‘uvpd’ R package (https://github.com/cpanse/uvpd/) and can be accessed at http://fgcz-ms-shiny.uzh.ch:8080/p2722-uvpd/. Its user interface is split into an input and output part. On the left, the input panel provides a selection for the input data, compounds, ionization mode and cut-off values for the relative and absolute mass errors of the precursor mass, precursor signal removal in the MS2 spectra and cluster ID. This selection then determines the respective output in the several tab panels on the right. These tabs provide data visualization and tables of the selected compound. Table 1 describes the output tabs and whether the selected filtering is applied to a given tab.

### 2.4. Higher-Throughout Comparison and Interpretation of UVPD and HCD Fragmentation Spectra

**Figure 3:**
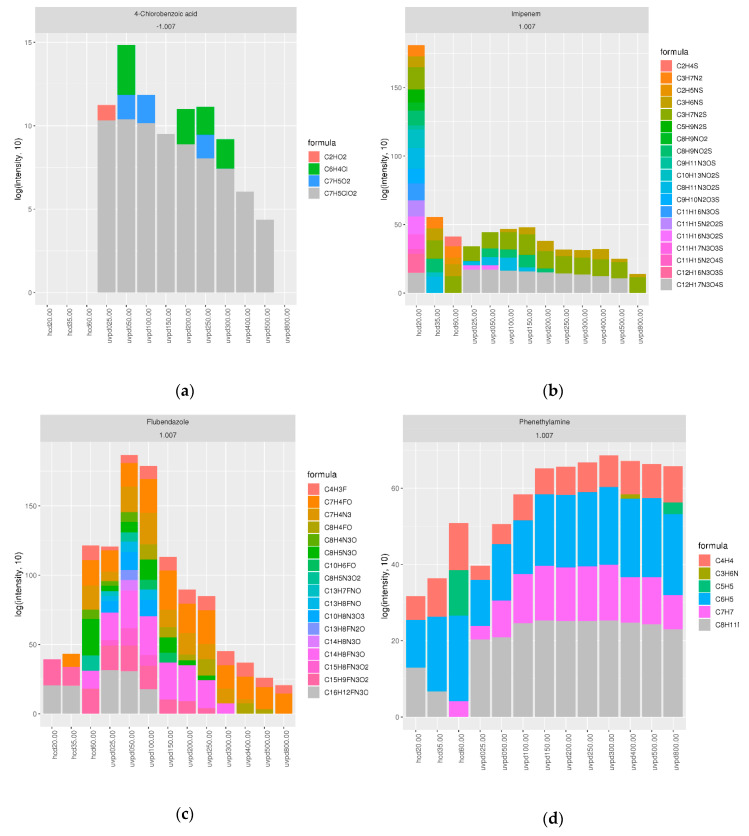
Fragmentation spectra annotation of the different ion types per fragmentation condition. Stacked bar plots from Shiny application output showing the summed intensities of the annotated fragments of (**a**) 4-chlorobenzoic acid; (**b**) imipenem; (**c**) flubendazole; (**d**) phenetylamine.

For a thorough comparison of UVPD and HCD fragmentation spectra, 46 selected water-relevant OMPs covering a wide chemical space were analyzed with LC-HRMS using UVPD with 25–800 ms reaction time, and HCD with 20, 35 and 60 CE. Eight of the compounds could not be detected with electrospray ionization (ESI), one eluted too early with reverse-phase (RP) LC for peak detection, one had an intensity below the cut-off threshold and two were not picked by the data analysis workflow due to a Na-adduct and Cl-salt, respectively (see Table S1). The remaining 34 compounds belonged to 11 different clusters, with one to four compounds per cluster. Their fragmentation behavior varied substantially and, based on fragmentation, four different groups of compounds could be distinguished (Table S1); such with poor fragmentation with both UVPD and HCD (Figure 3a), a preference for HCD (Figure 3b) or UVPD (Figure 3 c), or good fragmentation with both UVPD and HCD (Figure 3d). These groups, however, did not seem related to the cluster number.

The first group of compounds did not generate information-rich spectra, as illustrated in Figure 3a, which shows the summed intensities of the annotated fragments from HCD and UVPD spectra of 4-chlorobenzoic acid; poor fragmentation was observed for ibuprofen, 3-nitrophenol and 4-nitrophenol with both fragmentation techniques under all conditions, for 4-chlorobenzoic acid, 4-nitrophthalic acid and 5-nitroisophtalic acid in particular with HCD. In the case of 2-methyl-4-nitrophenol and gemfibrozil, spectra were still poor, but slightly better with HCD.

A second group of compounds showed a preference for HCD compared to UVPD (Figure 3b) either with increasing CEs, for instance 3-nitroindole, with low CEs, for instance imipenem at 20CE, with a specific CE, for instance 2-methyl-4-chlorophenoxyacetic acid (MCPA) at 35CE, or with all CEs, for instance N-Desmethyl Clarithromycin. In contrast, for another group of compounds, no fragmentation was observed with HCD, but good fragmentation with a range of UVPD reaction times (Figure 3c), for instance for benzocaine and to a lesser degree 4-nitroanthranilic acid. In the case of fenofibric acid, flubendazole and triadimenol good information rich spectra were generated with a range of UVPD reaction times, but only with HCD at 60 CE. 

A fourth group of compounds exhibited good fragmentation with both fragmentation techniques (Figure 3d). Some of these had an optimum at a specific fragmentation condition, for instance 2-amino-3-nitrobenzoic acid with UVPD at 50 ms, 3,5-Dinitrosalicylic acid with HCD at 35CE and UVPD at 50 ms, aflatoxin B2, dinoterb, epoxiconazole and JWH-250 with HCD at 60CE and 2-hydroxy-4-nitro-benzoic acid at 20 CE. Others fragmented well with a range of conditions, such as 2,4 Dinitrophenol, which showed more informative spectra with higher CEs, and 2-methoxy-4-nitrophenol and phenethylamine with higher HCD CEs and longer UVPD reaction times. Good fragmentation was observed for both (ranges of) UVPD and HCD conditions in the case of 2-methoxy-4,6-dinitrophenol, 4-hydroxy-3-nitrobenzenesulfonic acid, 4-nitrobenzenesulfonic acid and 5-nitrovanillin. In the case of nitrofurazone, more informative spectra were generated with UVPD.

Cluster numbers could not be related to these four broad groups of fragmentation behavior. For instance, while benzocaine and 4-Nitroanthranilic acid both fragmented well with UVPD and poorly with HCD, the other two compounds from cluster 13, piperacillin and 5-Nitroisophthalic acid, showed good fragmentation for both UVPD and HCD and poor fragmentation, respectively. Regarding cluster 11, aflatoxin b2 and fenofibric acid both exhibited information rich spectra at multiple different UVPD reaction times. However, gemfibrozil, a compound of the same cluster, did not fragment well with both UVPD and HCD. This lack of similar fragmentation behavior within a cluster could indicate that fragmentation behavior depends only on a few of the descriptors used for clustering.

In particular, UV absorbing compounds such as aromatic compounds, and compounds with double bonds are expected to fragment well with UVPD. Compound class information for each of the compounds that fragmented well with UVPD, including the lipids [3], flavonoids, phenylpropanoids and chalconoids [4,5] published previously could be utilized to predict UVPD fragmentation. Furthermore, if certain compounds of classes with good UV absorbance did not fragment well, further clustering within that compound class could be utilized to improve our understanding and ultimately the prediction of UVPD fragmentation behavior.

In the UVPD spectra, fragment ion intensities decreased with increasing UVPD reaction times when normalized to precursor intensity, as illustrated in Figure S2. Overall, UVPD fragmentation was beneficial for multiple compounds, often leading to a number of annotated fragments that were unique to the fragmentation technique. This complementarity of UVPD makes it an attractive addition to HCD that can be implemented in data-dependent decision trees during NTS data acquisition. Optimal UVPD reaction times depended on the compound, analogous to HCD where the optimal CE varied amongst compounds. Interestingly, in the HCD experiments, oftentimes, CEs higher (60 CE) and lower (20 CE) than the 35 CE routinely used in NTS experiments were needed to generate informative fragmentation spectra. This should be considered in future studies to increase the confidence of OMP identification, in particular when UVPD is not available.

### 2.5. NTS of a Meuse River Surface Water Sample

**Figure 4:**
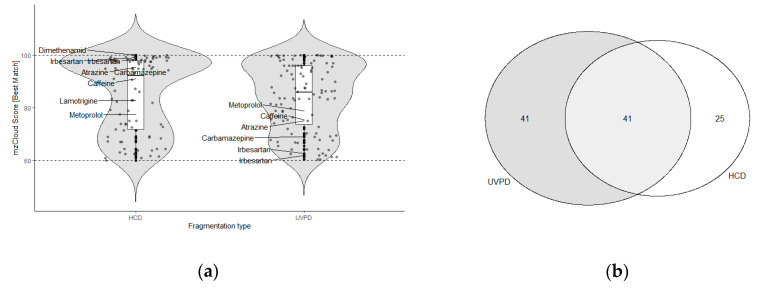
Comparison of HCD and UVPD NTS MS2 data of river Meuse water (**a**) mzCloud Score distribution. The scores of common water relevant compounds are labelled by compound name; (**b**) overlap in features annotations with an mzCloud score above 60.

To investigate the applicability of currently available spectral libraries and NTS workflows to UVPD data, a surface water sample from the river Meuse was acquired with HCD and UVPD fragmentation and analyzed using the NTS data analysis software Compound Discoverer (Thermo Fisher Scientific, San Jose, USA). This software enables suspect screening based on spectral matching with the spectral library mzCloud, which consists of collision-induced dissociation (CID) and HCD fragmentation spectra. The mzCloud score of a tentatively identified compound is a measure for the confidence of identification. It is based on the number of fragments that match the experimental and library spectra, with a score of 100 indicating a perfect match. Comparison of mzCloud scores of spectra acquired with HCD and UVPD showed that the overall score distribution was similar (no significant difference, see Appendix A), visualized in the combined box and violin plots in Figure 4a. However, individual compounds differed strongly in their scores. For instance, known water relevant OMPs on average showed a mzCloud score with UVPD fragmentation that was 15 points lower than the HCD score; atrazine scored 75.1 with UVPD versus 95.4 with HCD, caffeine 75.5 versus 91, carbamazepine 69.1 versus 96.6 and terbuthylazine 87.4 versus 98.6. UVPD spectra were matched with HCD library spectra of high CEs, i.e., 70 CE, 40 CE, 80 CE and 90 CE for the four different OMPs. In contrast, metoprolol showed a similar mzCloud score with UVPD, i.e., 78.9, when matched with an HCD 30 CE spectrum, compared to an HCD score of 77.6.

Half of the compound annotations with mzCloud in the NTS data with UVPD were not assigned in the HCD data (Figure 4b). The HCD data was manually checked for features with accurate mass and retention time matching these unique compounds. Information on whether these features were detected in the HCD data and their annotation is available in Table S2. Twenty-five features were detected in the HCD data, but had no mzCloud hit, and six were annotated with a different compound based on the mzCloud matching. Ten features were not detected. This is most likely due to differences in peak picking during data analysis. Manual inspection of the UVPD assignments showed that in most cases when there was no assignment in the HCD data, the annotated UVPD spectrum consisted of only a precursor signal and—if any—low intensity ions close to the noise cut-off (see SI Table S2 Compounds detected in UVPD NTS experiments). In these cases, the match was usually against a low energy CID or HCD library spectrum (CE10 to 20) that also only contained the precursor. Consequently, these can likely be false positive assignments. The high scores for matches based on the precursor signal alone are problematic. In future studies, more appropriate scoring algorithms should be considered.

In contrast, the assignments where multiple fragments were matched, i.e., 1-methylbenzotriazole with 15, 3-hydroxyfluorene with 11, acetyl norfentanyl with three, mandipropamid with seven and metolachlor with 17 fragments, were all based on high energy HCD library spectra (60 to 130 CE) except for 3-hydroxfluorene (20 CE). This was in correspondence with the assignments of known water relevant OMPs, and indicates that UVPD-induced fragmentation pathways in these molecules resemble those of higher energy HCD. As routinely lower HCD CEs are applied, i.e., 20–50 CE, this can explain why these assignments were only made in the case of UVPD fragmentation, emphasizing the benefit of this alternative fragmentation technique and/or higher CEs for NTS based identification of OMPs. Moreover, UVPD annotations could be used to exclude false positive annotations of sparse HCD MS2 spectra (precursor only matches) and vice versa. While HCD spectral libraries proved to be of (limited) use for UVPD spectral annotation, for the routine implementation of UVPD data in NTS workflows, spectral libraries need to be extended with UVPD spectra.

## 3. Materials and Methods

### 3.1. Selection of Reference Standards Through Clustering

Selection criteria for OMPs to be fragmented with UVPD and HCD included their relevance for the water sector and a good coverage of the chemical space, as the compound structures were expected to affect fragmentation. To select water relevant OMPs, 4000 compounds were randomly selected from the NORMAN Substance Database, which is compiled of multiple suspect lists relevant for environmental monitoring (SusDat, https://www.norman-network.com/nds/susdat/). To select compounds with diverse chemical structures, these compounds were clustered [9]. To this end, the Simplified molecular-input line-entry systems (SMILES) of each compound were parsed and configured for atom typing and isotoping using the R package rcdk [10]. Next, for each compound, the extended fingerprint, a binary vector of 1024 dimensions, was extracted. A k-means clustering was conducted of the computed Tanimoto Distance matrix between all pairs of fingerprints [11]. The optimal number of clusters was determined by the elbow method [12]. To investigate molecular discrimination by the clusters, we trained a self-organizing map (SOM) [13] as a complementary approach. The SOM grid was initialized with 10 × 10 nodes. Each fingerprint selected in the training phase was colored by the corresponding k-means cluster ID for visualization. The entire *in silico* data analysis was performed using R version 3.5.1 to 4.0.1 running on Linux, Windows and MacOSX systems [14]. All code snippets are available as an R package through https://github.com/cpanse/uvpd/.

### 3.2. LC-HRMS Analysis with UVPD Fragmentation

Selected reference compounds listed in SI Table S2 were prepared in ultra-pure water with a final concentration of 10 µg/L. The surface water (SW) sample was collected from the river Meuse, the Netherlands, 16.666× concentrated using Oasis-HLB SPE columns-based extraction and diluted 50× for the LC-HRMS analysis. The internal standards (IS) atrazine-d5 (CDN isotopes, Pointe-Claire, Quebec, Canada), benzotriazole-d4 and bentazon-d6 (LGC Standards, Wesen, Germany) were added to the SW sample to a final concentration of 1 µg/L. Samples were filtered using 0.2 µm Phenex^TM^-RC 15 mm Syringe Filters (Phenomenex, Torrance, USA) prior to analysis. Blank samples were prepared correspondingly, through spike-in of IS to ultra-pure water followed by filtration. In total, 100 μL of sample were injected into the LC-HRMS.

Compounds were analyzed using reverse phase (RP) LC-HRMS/MS with a Vanquish Horizon UHPLC system (Thermo Fisher Scientific, San Jose, CA, USA) coupled to an Orbitrap Fusion Lumos equipped with ultraviolet photodissociation (UVPD) and the acquisition software AcquireX (Thermo Fisher Scientific, San Jose, CA, USA). An XBridge BEH C18 XP column (150 mm × 2.1 mm I.D., particle size 2.5 μm, Waters, Etten-Leur, The Netherlands) was used in combination with a 2.0 mm × 2.1 mm I.D. Phenomenex SecurityGuard Ultra column (Phenomenex, Torrance, CA, USA), at a temperature of 25 °C. The LC gradient started with 5% acetonitrile, 95% water and 0.05% formic acid (*v*/*v*/*v*), increased to 100% acetonitrile, 0.05% formic acid in 25 min and then remained constant for 4 min. The flow rate was 0.25 mL/min. 

For the reference standards, fragmentation spectra were acquired using targeted methods with mass triggers. The fixed collision energies (CEs) 20, 35 and 60 were used for HCD fragmentation, and UVPD reaction times ranging from 25 to 800 ms for UVPD fragmentation. The full scan mass range was 100–800 *m*/*z* with 120k resolution at FWHM for the MS1 scans, and 50–500 *m*/*z* with 15k resolution at FWHM for the MS2 scans (due to a corrupted data file, the disinfection by-products data with 100 ms and 25 0 ms UVPD reaction time is lacking in the data set).

For the SW sample, NTS analyses were performed with data dependent acquisition (ddA), using the AcquireX deepscan functionality that ensures MS2 scans are acquired for most features, Top Speed and 35 CE for the HCD and 100 and 150 ms reaction time for the UVPD experiments. The full scan mass range was set at 80–1300 *m/z* with 120k resolution, the MS2 at 50–500 *m/z* with 15k resolution.

### 3.3. Manual Annotation of Fragmentation Spectra

Thermo Fisher Scientific raw files were viewed with Thermo Xcalibur Browser (Thermo Fisher Scientific, San Jose, CA, USA). MS2 peak lists of HCD and UVPD fragmentation spectra were exported and used for fragment annotation with the MetFrag web tool (https://msbi.ipb-halle.de/MetFragBeta/).

### 3.4. An R-Based LC-HRMS Data Analysis Workflow to Explore Novel Fragmentation Techniques

Fragment ions of the selected reference standard compounds were predicted with tree depth 1 and 2, using the R package MetFrag [7] in a preprocessing step. Charge configurations were derived for the predicted singly charged fragments [M]^+^, [M + H]^+^ and [M + 2H]^+^ and [M]^−^, [M − H]^−^ and [M − 2H]^−^ for the positive and negative ionization mode, respectively. The predicted fragment ions were stored and made available as a dataset in the R package UVPD.

Thermo Fisher Scientific raw files were processed with the R package rawDiag [15]. The in profile mode recorded data were centroided using the centroid method of the R package protViz (https://CRAN.R-project.org/package=protViz, [16]). For all compounds measured in all fragmentation modes, the retention time (RT), the area under the curve (AUC) of the APEX extracted ion chromatography (XIC) of the protonated and deprotonated precursor species, i.e., [M + H]^+^ and [M − H]^−^ of the selected reference standard compounds, the master intensity and the total ion count (TIC) were determined (see Figure S3). The *m/z* of the [M + H]^+^ and [M − H]^−^ were calculated based on the compound SMILES. The top three highest intensity spectra of each reference compound per fragmentation mode were used to assess the performance of the different fragmentation modes. The peaks of the centroided fragmentation spectra were annotated with the previously predicted fragment ions if the match was within a given mass window. A default cut-off value for fragment matching was set to 1 Da, further refinement can be made in the Shiny application (1–100 ppm, 10e–4 to 0.5 Da). The default values in the Shiny application are relative and absolute cut-off of 10 ppm and 0.02 Da, respectively. These are also the tolerances used throughout the manuscript.

The quantitative, e.g., MS1 derived XIC and master intensity, and MS2 derived TIC and fragment intensities, and qualitative fragmentation data were joined by the raw file name and the scan number. To compare fragment ion annotation qualitatively and quantitatively across all compounds and fragmentation modes, we implemented three different scores:

(1) $$Score\;1=\frac{n_{exp\;frags\;matched}}{n_{theor\;frags}}$$

(2) $$Score\;2=\frac{n_{exp\;frags\;matched}}{n_{exp\;frags}}$$

(3) $$Score\;3=\frac{{int}_{exp\;frags\;matched}}{{int}_{\mathrm{MS}1\;precursor}}$$

where *exp frags* are the experimentally detected fragments, *exp frags* matched the experimentally detected fragment ions that could be matched to the *in silico* predicted, and *theor frags* the *in silico* predicted theoretically possible fragment ions. *n* indicates the number of fragments and *int* their intensity.

All data and results are visualized and can be interactively accessed in the R shiny application provided with the uvpd R package and through http://fgcz-ms-shiny.uzh.ch:8080/p2722-uvpd/. The entire workflow is shown in Figure S2.

### 3.5. NTS Data Analysis

NTS data were processed with Compound Discoverer 3.1 (Thermo Fisher Scientific, San Jose, CA, USA) for peak picking, componentization and suspect screening using the spectral library mzCloud. The output feature list, i.e., a table with accurate mass/retention time pairs (features) and their intensity, information on whether an MS2 spectrum was acquired for a given feature and the mzCloud spectral matching scores were imported into R Studio for further data analysis and visualization. R version 3.6.3. and R-Studio version 1.1.463 were used for the data analysis [14,17].

## 4. Conclusions

Combining the novel fragmentation technique UVPD and cheminformatics tools, we showed the potential of UVPD for structural elucidation of water-relevant OMPs in NTS data. Based on the two complementary methods k-means clustering and SOMs, a set of OMPs could be selected that was representative for the water cycle and a wide chemical space. An R-based LC-HRMS data analysis workflow and interactive interface for data visualization was developed to investigate UVPD fragmentation of these OMPs in a high-throughput manner.

Information-rich UVPD fragmentation spectra were achieved for 62% of the examined OMPs, in 15% of the cases also for OMPs that fragmented poorly with HCD. For 26% of the OMPs, neither fragmentation technique generated informative spectra; the remaining 12% HCD spectra were information-rich. UVPD and HCD generated both unique as well as overlapping fragments, demonstrating that some fragmentation pathways are specific to the respective fragmentation methods, while others seem to be more generic. These unique fragments provided additional information for structural identification complementary to HCD spectra. Based on these results, implementation of UVPD as a second fragmentation option in data dependent decision trees during NTS data acquisition is an attractive strategy to improve the confidence in OMP identification.

Analysis of NTS UVPD data with existing NTS software and the spectral library mzCloud enables annotation of features in the UVPD data using HCD library spectra of high CEs. For the routine implementation of UVPD fragmentation in NTS workflows, however, databases need to be extended with UVPD spectra, which would allow the full potential of this novel fragmentation technique to be exploited.

## Figures and Tables

**Table 1:** Description of Shiny application output panels and applied filtering parameters.

Tab Panel	Description	Selected Filter Option Applied to the Tab				
		Compound	Remove Precursor Items	(+/−) Ion Type	Ppm Error Cut-Off	Absolute Error Cut-Off
stacked fragments	1) Bivariate scatterplots of scores 1, 2 and 3 per fragmentation mode. 2) Two stacked bar charts of the logarithmically transformed fragment ion intensities of the matched fragment ions and types, respectively, per fragmentation mode. 3) Bivariate scatterplots of the total ion count (TIC) of the MS2 spectrum and the corresponding master intensity for the three most abundant master intensities of each raw file per fragmentation mode. 4) Boxplots of the absolute error distribution (in Dalton) per fragmentation mode.	X	X	X	X	X
summary	1) Statistics of the overall data and the applied filter setting. 2) Frequency value per fragmentation mode. 3) Histograms of ppm and absolute error distribution over the entire data set and selected compound, including a maximum-likelihood fitting, assuming an underlying normal distribution.	X	X	X	X	X
ms2	1) Table of detected fragment ions and ion types. 2) Fragmentation spectra per fragmentation mode.	X	X	X	X	X
data	All quantitative and qualitative data.	X	X	X	X	X
scores	1) Scores 1, 2 and 3. 2) Plots of the scores.		X	X	X	X
frequencies	Downloadable frequency table, per compound and fragmentation type		X	automatic	X	X
predicted ion	*In silico* predicted, i.e., theoretical fragment ions predicted with ‘metfRag: frag.generateFragments’	X				
help	Help page					

## Supplementary Materials

The following are available online, Figure S1: Negative ionization mode model OMPs (a) gemfibrozil and (b) sucralose; Figure S2: Fragment intensity decreases with increasing UVPD reaction times. Total ion counts (TIC) for a given fragmentation mode versus master intensity; Figure S3: Data analysis and visualization workflow, Table S1: List of reference standards; Table S2: Compounds detected in UVPD NTS experiments.

## Availability

Used code snippets available at https://github.com/cpanse/uvpd/; Shiny app available at http://fgcz-ms-shiny.uzh.ch:8080/p2722-uvpd/; LC-HRMS raw data of set of reference standards available at https://doi.org/10.5281/zenodo.4001653.

## Appendix A

There was no significant difference between mzCloud scores of NTS data with HCD and UVPD fragmentation:

t.test(data$mzCloud.Best.Match ~ data$frag)

Welch Two Sample t-test

data: data$mzCloud.Best.Match by data$frag

*t* = 0.44183, df = 177.56, *p*-value = 0.6591

alternative hypothesis: true difference in means is not equal to 0

95 percent confidence interval:

−2.969521 4.682821

sample estimates:

mean in group HCD mean in group UVPD

84.99091 84.13426
