# Supplementary figures and images for: Ultraviolet Photodissociation for Non-Target Screening-Based Identification of Organic Micro-Pollutants in Water Samples
Source: Molecules. 2020 Sep 12;25(18):4189. doi: 10.3390/molecules25184189 (PMC7570901; doi:10.3390/molecules25184189)

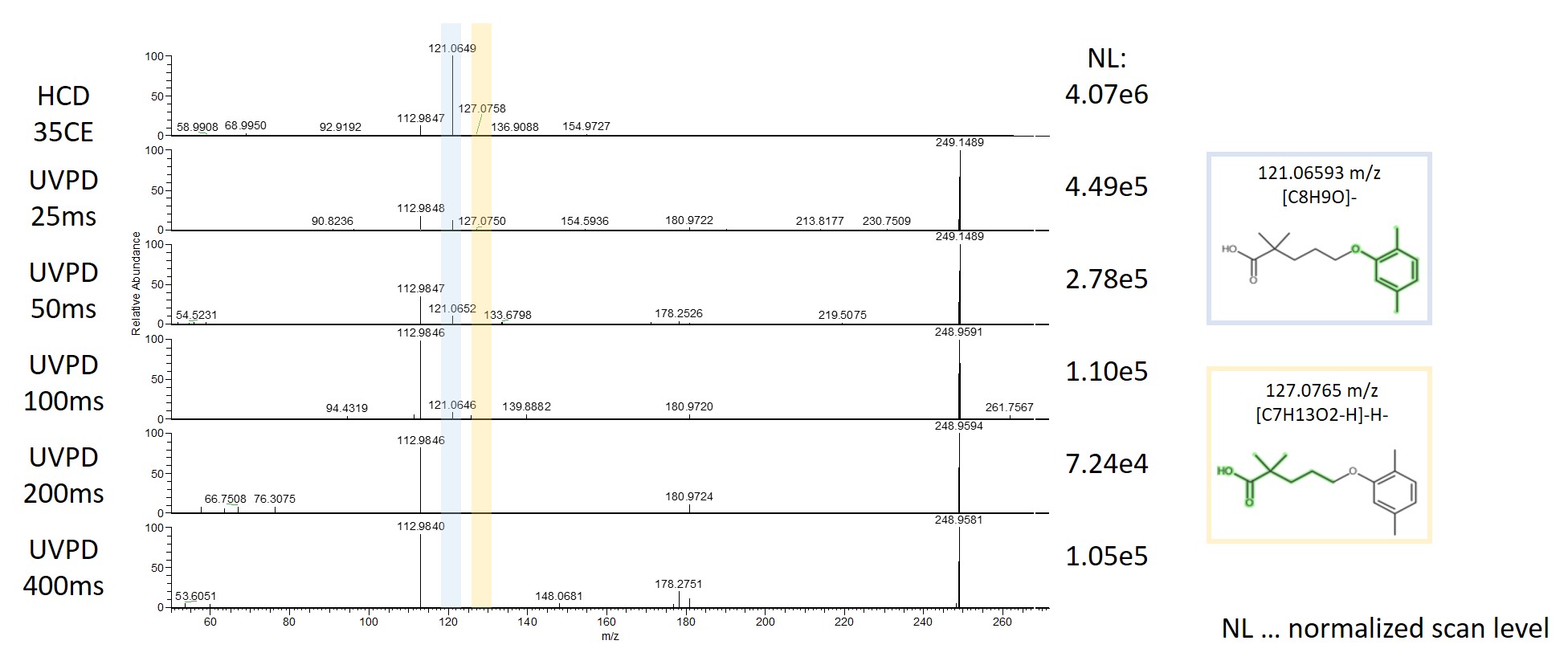

Supplement: Supplementary file 1 [file molecules-25-04189-s001.zip › SFigure1a_gemfibrozil_rev.jpg]

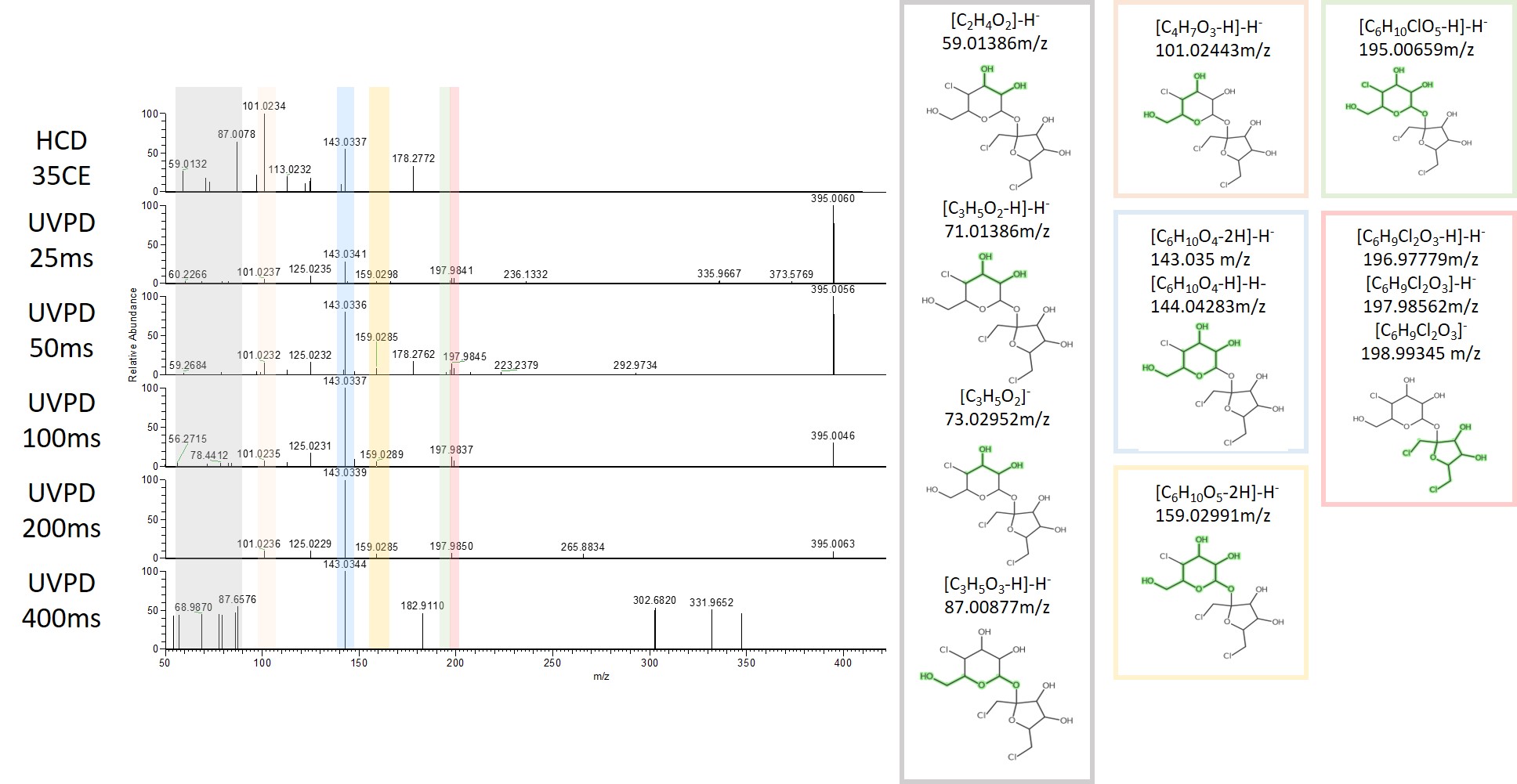

Supplement: Supplementary file 1 [file molecules-25-04189-s001.zip › SFigure1b_sucralose_rev.jpg]

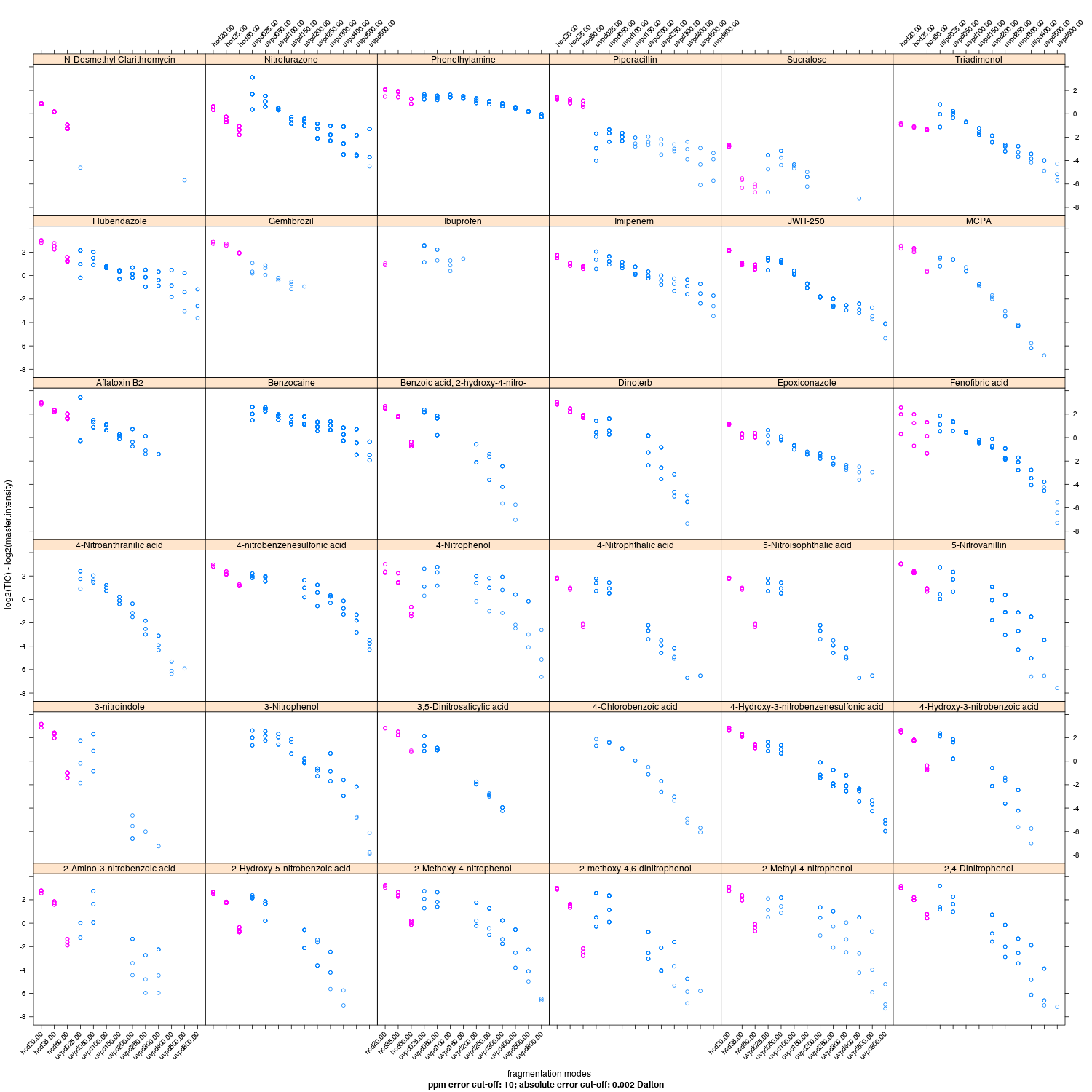

Supplement: Supplementary file 1 [file molecules-25-04189-s001.zip › SFigure2_TICvsMastIntt.png]

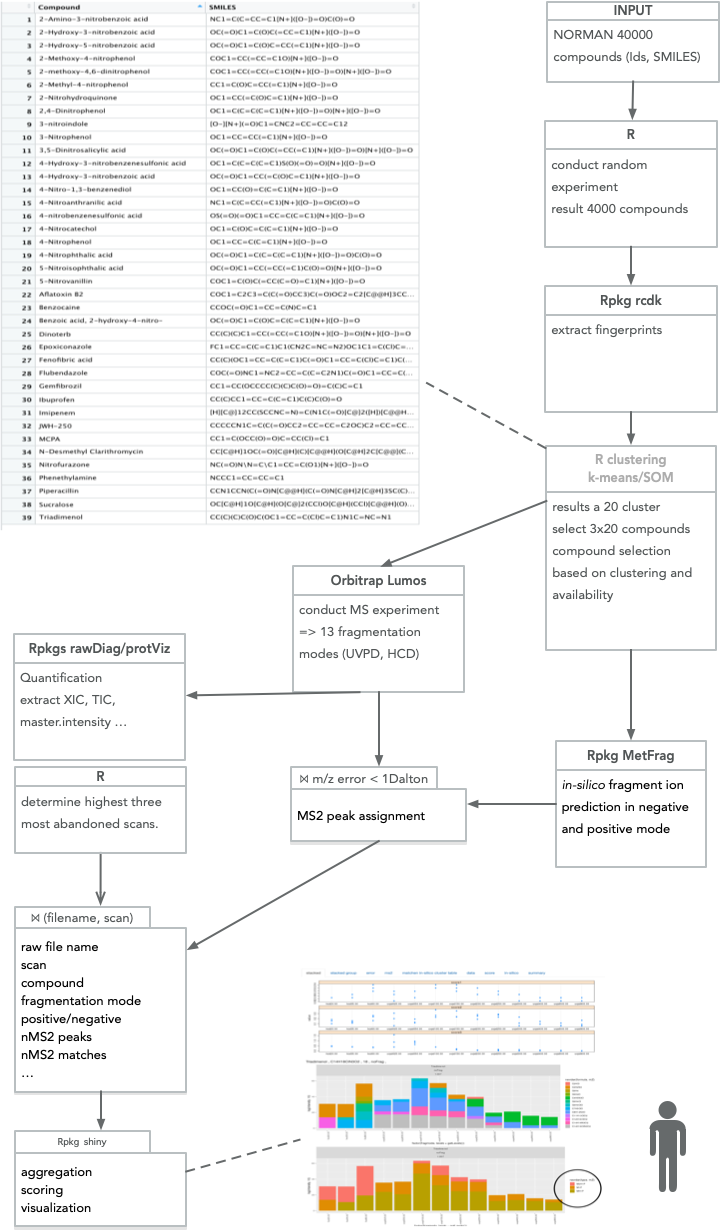

Supplement: Supplementary file 1 [file molecules-25-04189-s001.zip › SFigure3_DataAnalworkflow_rev.png]
